# Developing an agenda for the decolonization of global health

**DOI:** 10.2471/BLT.23.289949

**Published:** 2023-12-08

**Authors:** David McCoy, Anuj Kapilashrami, Ramya Kumar, Emma Rhule, Rajat Khosla

**Affiliations:** a International Institute for Global Health, United Nations University, Hospital Canselor Tuanku Muhriz UKM, Jalan Yaacob Latif, Bandar Tun Razak, Cheras, 56000 Kuala Lumpur, Malaysia.; b School of Health and Social Care, Essex University, Colchester, England.; c Faculty of Medicine, University of Jaffna, Jaffna, Sri Lanka.

## Abstract

Colonialism, which involves the systemic domination of lands, markets, peoples, assets, cultures or political institutions to exploit, misappropriate and extract wealth and resources, affects health in many ways. In recent years, interest has grown in the decolonization of global health with a focus on correcting power imbalances between high-income and low-income countries and on challenging ideas and values of some wealthy countries that shape the practice of global health. We argue that decolonization of global health must also address the relationship between global health actors and contemporary forms of colonialism, in particular the current forms of corporate and financialized colonialism that operate through globalized systems of wealth extraction and profiteering. We present a three-part agenda for action that can be taken to decolonize global health. The first part relates to the power asymmetries that exist between global health actors from high-income and historically privileged countries and their counterparts in low-income and marginalized settings. The second part concerns the colonization of the structures and systems of global health governance itself. The third part addresses how colonialism occurs through the global health system. Addressing all forms of colonialism calls for a political and economic anticolonialism as well as social decolonization aimed at ensuring greater national, racial, cultural and knowledge diversity within the structures of global health.

## Résumé

Le colonialisme, qui implique la domination systémique de terres, de marchés, de peuples, de ressources, de cultures ou d’institutions politiques dans le but d’exploiter, de détourner et d’extraire des richesses et des ressources, affecte la santé de nombreuses manières. Ces dernières années, la décolonisation de la santé mondiale a suscité un intérêt croissant, l’accent étant mis sur la correction des déséquilibres de pouvoir entre les pays à revenu élevé et les pays à faible revenu, ainsi que sur la remise en question des idées et des valeurs de certains pays riches qui façonnent la pratique de la santé mondiale. Nous soutenons que la décolonisation de la santé mondiale doit également aborder la relation entre les acteurs de la santé mondiale et les formes contemporaines de colonialisme, en particulier les formes actuelles de colonialisme d’entreprise et de colonialisme financiarisé qui opèrent par des systèmes mondialisés d’extraction de richesses et de profits. Nous présentons un programme d’action en trois parties destiné à décoloniser la santé mondiale. La première partie porte sur les asymétries de pouvoir existant entre les acteurs de la santé mondiale des pays à hauts revenus et historiquement privilégiés et leurs homologues des pays à faibles revenus et marginalisés. La deuxième partie concerne la colonisation des structures et des systèmes de la gouvernance mondiale de la santé elle-même. La troisième partie traite de la manière dont le colonialisme se manifeste à travers le système de santé mondial. La lutte contre toutes les formes de colonialisme nécessite un anticolonialisme politique et économique ainsi qu’une décolonisation sociale visant à garantir une plus grande diversité nationale, raciale, culturelle et des connaissances au sein des structures de la santé mondiale.

## Resumen

El colonialismo, que implica la dominación sistémica de tierras, mercados, pueblos, bienes, culturas o instituciones políticas para explotar, apropiarse indebidamente y extraer riqueza y recursos, afecta a la salud de muchas maneras. En los últimos años ha crecido el interés por descolonizar la salud mundial, en particular para corregir los desequilibrios de poder entre los países de ingresos altos y los de ingresos bajos, y para cuestionar las ideas y los valores de algunos países ricos que influyen en la práctica de la salud mundial. Sostenemos que la descolonización de la salud mundial también debe abordar la relación entre los actores de la salud mundial y las formas contemporáneas de colonialismo, en especial las formas actuales de colonialismo corporativo y financiarizado que operan a través de sistemas globalizados de extracción de riqueza y especulación. Presentamos un programa de acción dividido en tres partes para descolonizar la salud mundial. La primera parte se refiere a las asimetrías de poder que existen entre los actores de la salud mundial procedentes de países de ingresos altos e históricamente privilegiados y sus homólogos de entornos de ingresos bajos y marginados. La segunda parte se refiere a la colonización de las estructuras y sistemas de la propia gobernanza de la salud mundial. La tercera parte aborda cómo se produce el colonialismo a través del sistema sanitario mundial. Abordar todas las formas de colonialismo exige un anticolonialismo político y económico, así como una descolonización social destinada a garantizar una mayor diversidad nacional, racial, cultural y de conocimientos dentro de las estructuras de la salud mundial.

## ملخص

ينطوي الاستعمار على السيطرة المنظمة على الأراضي، أو الأسواق، أو الشعوب، أو الأصول، أو الثقافات، أو المؤسسات السياسية، وذلك بغرض استغلال الثروات والموارد، وسلبهما واستخراجهما، وهو ما يؤثر على الصحة بعدة طرق. في السنوات الأخيرة، تزايد الاهتمام بإنهاء سيطرة الاستعمار على الصحة العالمية مع التركيز على تصحيح اختلال توازن القوى بين الدول مرتفعة الدخل والدول منخفضة الدخل، وعلى الأفكار والقيم الصعبة لبعض الدول الغنية التي تشكل ممارسة الصحة العالمية. نحن نزعم أن إنهاء سيطرة الاستعمار على الصحة العالمية يجب أن يتعامل أيضًا مع العلاقة بين الأطراف الفاعلة في مجال الصحة العالمية، والأشكال المعاصرة للاستعمار، وبخاصة الأشكال الحالية لاستعمار الشركات والاستعمار المالي الذي يعمل من خلال أنظمة عولمة لاستخراج الثروة وتحقيق الأرباح. إننا نقدم أجندة عمل مكونة من ثلاثة أجزاء يمكن اتخاذها لإنهاء سيطرة الاستعمار على الصحة العالمية. ويتعلق الجزء الأول بعدم تناسق القوى الموجود بين الأطراف الفاعلة في مجال الصحة العالمية من الدول مرتفعة الدخل، والدول البارزة تاريخياً، ونظيراتها ذات الأوضاع منخفضة الدخل والمهمشة. ويتعلق الجزء الثاني بسيطرة الاستعمار على هياكل وأنظمة إدارة الصحة العالمية نفسها. ويتناول الجزء الثالث كيفية حدوث الاستعمار من خلال النظام الصحي العالمي. إن التعامل مع جميع أشكال الاستعمار يدعو إلى مواجهة الاستعمار السياسي والاقتصادي، وكذلك إنهاء سيطرة الاستعمار الاجتماعي بهدف ضمان قدر أكبر من التنوع الوطني، والعرقي، والثقافي، والمعرفي داخل هياكل الصحة العالمية.

## 摘要

殖民主义在许多方面影响人们的健康，主要涉及对土地、市场、人民、资产、文化或政治机构的系统性统治，以剥削、盗用和榨取财富和资源。近年来，人们对全球卫生去殖民化的关注程度愈来愈高，主要关注矫正高收入和低收入国家之间的权力失衡，挑战一些富裕国家的思想和价值观，这些思想和价值观影响着全球的卫生实践。我们认为，全球卫生去殖民化还必须处理全球卫生行动机构与当代形式殖民主义之间的关系，特别是通过全球化的财富攫取和暴利体系运作的当前形式的企业和金融化殖民主义。我们提出了一个由三部分组成的行动议程，用来实现全球卫生去殖民化。第一部分涉及来自高收入和历史上享有特权国家的全球卫生行动机构与低收入和边缘化国家的同行之间存在的权力不对称。第二部分涉及全球卫生治理结构和体系本身的殖民化。第三部分讨论殖民主义是如何通过全球卫生系统发生的。解决一切形式的殖民主义需要政治和经济反殖民主义以及社会去殖民化，以确保全球卫生结构内更大的民族、种族、文化和知识多样性。

## Резюме

Колониализм, который подразумевает системное господство над землями, рынками, народами, ресурсами, культурами или политическими институтами с целью эксплуатации, присвоения и извлечения богатств и ресурсов, во многом оказывает влияние на здоровье. В последние годы возрос интерес к деколонизации глобального здравоохранения с акцентом на устранение дисбаланса сил между странами с высоким и низким уровнем доходов и на оспаривание идей и ценностей некоторых стран с высоким уровнем жизни, которые определяют практику глобального здравоохранения. Авторы статьи утверждают, что деколонизация глобального здравоохранения должна также затрагивать отношения между субъектами глобального здравоохранения и современными формами колониализма, в частности современными формами корпоративного и финансированного колониализма, которые действуют через глобализованные системы извлечения богатства и спекуляции. В этой статье представлена программа действий, состоящая из трех частей, которая может быть использована для деколонизации глобального здравоохранения. Первая часть посвящена асимметрии власти, существующей между субъектами глобального здравоохранения из стран с высоким уровнем доходов и исторически привилегированных стран и их коллегами из стран с низким уровнем доходов и маргинализированных слоев населения. Вторая часть касается колонизации структур и систем глобального управления здравоохранением. В третьей части рассматриваются способы проявления колониализма в глобальной системе здравоохранения. Борьба со всеми формами колониализма требует проведения политического и экономического антиколониализма, а также социальной деколонизации, направленной на обеспечение большего национального, расового, культурного и информационного разнообразия в структурах глобального здравоохранения.

## Introduction

Medicine and public health have always been shaped by social and political values,^1^ as evident in efforts to redress social inequities in health, struggles to realize health as a universal human right and, more recently, in calls to decolonize global health.^2^ Indeed, reviewing global health through the lens of colonialism or coloniality provides an opportunity to consider the role of power, injustice and exploitation in the practice of global health. However, much of the current interest in decolonizing global health has been focused on the domination of global health by actors and institutions from high-income and historically privileged countries, and the resulting hierarchies in knowledge production. Less attention has been paid to new forms of colonialism and how the complex of global health structures and systems may itself be a channel for extractive colonial practices. In this paper, we provide a more comprehensive discussion of colonialism and its manifestations in global health, and offer a conceptual framework to identify the different sites and forms of colonial practice and how these practices can be countered.

## Understanding colonialism

We define colonialism as one group of people having the power to dominate, subjugate and/or exploit another group or groups of people, thereby enabling the misappropriation and extraction of resources in a large-scale and systematic manner. These resources may be human resources in the form of cheap, indentured or enslaved labour; natural resources such as fertile land, minerals, oil and genetic material from plants and microorganisms; and intangible resources such as data and knowledge. Colonialism is most commonly associated with imperialism and the conquest, control or occupation of foreign lands. Thus, decolonization is most often used to describe the physical withdrawal of a foreign power from its colonies and the establishment of new sovereign states. However, one must understand that colonialism manifests in different ways and that it is a contemporary phenomenon.

Ghana’s first President, Kwame Nkrumah, coined the term neocolonialism to describe the continued extraction of resources and wealth from newly independent states by their former rulers and other foreign powers.^3^ This neocolonialism is achieved through indirect political and economic control – often backed with the use or threat of use of military force – including the use of financial and economic power to buy assets and capture markets, and the control of the institutions of global economic governance to establish advantageous monetary, trade and investment systems.^3^ Neocolonialism also involves powerful external actors working with post-independence governments and elites to continue systems of subjugation and exploitation, including systems established by their former colonial rulers. The large net outflow of resources and wealth from independent states in sub-Saharan Africa to beneficiaries in high-income countries is evidence of the scale and power of neocolonial forms of exploitation.^4^^,^^5^

Colonial oppression and exploitation can also manifest internally within the borders of the modern nation state, including states that were former colonies. For example, having freed itself of British rule, the United States of America continued the colonization of indigenous native Americans and enslavement of millions of Africans. Today, in many countries, indigenous or minority communities are subjected to unjust political and economic systems and oppressive and exploitative arrangements that could be considered colonial.

A key feature of colonialism is its relationship with capitalism. Finance capital helps create and consolidate the economic, military and technological power of colonial actors. Furthermore, through its need to constantly seek out new sources of profit, capitalism drives the impulse to colonize. Capitalist colonial structures often involve governmental and private actors working together. British colonial rule in India and North America, for example, developed through evolving interactions between private corporations such as the East India and Hudson Bay companies and the British state.^6^ Today, much contemporary colonialism is organized around powerful private financial institutions and transnational corporations with control over large parts of the global economy.^7^ Underpinning this control has been a growth in the volume and mobility of financial capital, and the global integration of markets and supply chains under a largely neoliberal policy model, mostly implemented through global economic institutions such as the International Monetary Fund and World Trade Organization and many other multilateral, plurilateral and bilateral trade and investment agreements.^8^

Central to the various forms of globalized financial and corporate colonialism present today are: financial deregulation; the strengthening of intellectual property rights which may be equated to a colonization of knowledge; and the enablement of tax avoidance through the deregulation of transnational corporate activity and the tolerance of secretive banking regimes which allow public funds to be misappropriated.^9^^,^^10^ Financialization and the privatization of societal institutions that were previously considered public (e.g. education, health care, public utilities such as water and sewerage, and even prisons and policing) have further expanded opportunities for wealth extraction and accumulation.^11^ The effects of contemporary colonialism are considerable. As an ever-increasing share of profits across all economic sectors are enjoyed by a small transnational elite, workers across the world are experiencing falling wages, working conditions are deteriorating and becoming increasingly precarious, and hundreds of millions of people remain in extreme poverty.^12^ By one estimate, 10 men own more wealth than the poorest 3.1 billion people in the world.^12^

The pattern of wealth extraction today still mirrors the previous exploitative relationships between colonizing countries and their former colonies. However, today’s globalized political economy has also altered the geography of colonialism. Contemporary colonialism is marked by the growth of extreme wealth and poverty in both rich and poor countries alike, and the emergence of a globalized class structure that includes an elite that transcends national, racial and religious identities, and rising numbers of impoverished people in high-income countries. The growing digitalization of the global economy also underscores the changing geographic contours of colonialism. Although the physical colonization of land^13^^,^^14^ and other natural resources is still important, the virtual digital spaces through which so much economic activity now occurs is a new and important site of colonialism.^15^ Great wealth is now extracted through the rent-seeking practices of monopolistic technology (tech) companies with control over e-commerce platforms and large data sets of individual preferences and behaviours. In what has been labelled as surveillance capitalism, many people are now exposed to manipulative and targeted extractive marketing, as well as to unprecedented levels of intrusive surveillance and monitoring.^16^

Fundamental to all forms of colonialism is the use of ideas and narratives by colonialists to enable and legitimize colonial practices. Racist ideas of European moral and cultural superiority in public life, and the portrayal of colonial subjects as inferior, were powerful forces of European colonialism and central to its most brutal manifestations, including the extermination of indigenous populations and the trans-Atlantic slave trade. Racism and other forms of discrimination continue to shape exploitative and oppressive relationships today, as well as paternalistic approaches to development and humanitarian work.^17^ However, contemporary anticolonial struggles must also challenge economic ideas and narratives that are used to sustain and legitimize the extractive and exploitative practices of today’s globalized colonialism.

These ideas and narratives include: false or exaggerated claims, delivered through powerful corporate media and networks of well-funded lobbyists, think tanks and research groups, about the virtues and benefits of unregulated markets and expanded intellectual property rights; manufactured misinformation about global warming; and the excessive devotion to technological innovation as a means to tackling the problems of poverty without any need for redistribution of resources or sociopolitical change.^18^^,^^19^ Furthermore, just as European colonizers used missionary doctors and teachers to portray themselves as saviours, corporate social responsibility and billion-dollar private foundations are used to portray today’s economic elites as benevolent wealth creators or entrepreneurial problem-solvers for the world.^20^ Such narratives not only hide the true nature of contemporary colonialism, but also often reinforce power asymmetries by promoting proprietary knowledge and technologies as solutions to social and political problems.

The complex intersection between colonialism and gendered patterns of subjugation and exploitation also needs specific mention. All colonial powers typically impose their sociocultural norms and beliefs on colonized peoples. European colonialism, for example, imposed a particular form of patriarchy (including binary norms related to sex, gender and sexuality) through laws and practices that subverted local customs in some places, and which were used to control the bodies of marginalized groups in society.^21^ In more recent times, by contrast, certain forms of feminism that have arisen in some high-income countries have been used in low-income countries to address patriarchy and gender inequality, but sometimes in ways that may be seen as a form of cultural imperialism.^21^ At the same time, under today’s globalized capitalist systems, gendered patterns of economic exploitation and their intersection with class and race are seen in the concentration of precarious, low-paid and sometimes dangerous work within the formal and informal economic sectors among women of colour and women in low-income countries.^21^Additionally, an unfair and disproportionate amount of unpaid care work is being done by women everywhere.^22^

A comprehensive conceptualization of colonialism must include an ecological dimension in the age of the Anthropocene (a new unit of geological time used to describe the most recent period in the Earth’s history during which human activity has substantially affected the planet's natural and biophysical systems). Historically, colonialism and the underlying forces of capitalism have been associated with the plunder of natural resources and destruction of the natural environment, the effects of which were often disastrous to indigenous and local communities.^23^ Furthermore, the unequal contribution to greenhouse gas emissions and disproportionate negative impact of global warming on low-income countries and populations represent an ongoing injustice rooted in colonial history.^24^^,^^25^ Indeed, the prospect of future generations living on a planet stripped of the key ecosystem support required for human civilization may also be viewed as a form of intergenerational colonialism involving unjust and extractive relationships between populations in different periods of time.^26^ Pertinently, many of the indigenous cultures and knowledge systems that have been destroyed by colonialism instilled the idea that all generations have a custodial duty to protect the natural environment for future generations.^26^

## Colonialism, medicine and health

Colonialism has shaped medicine and public health in various ways. For example, tropical medicine and tropical medicine institutes (e.g. the Institute of Tropical Medicine in Antwerp, and the London School of Hygiene and Tropical Medicine) were established to protect colonial personnel, maintain the productivity of native workers and aid imperial expansion. Additionally, colonialism has included: the use of a false medical science to legitimize claims of white superiority; the imposition of biomedicine to the detriment of indigenous systems of health care; the deployment of missionary medicine to cultivate an image of colonial benevolence; the misappropriation of local knowledge and traditional remedies; and the subjection of colonized populations to unethical medical experimentation, and vaccine and drug trials.^27^^–^^31^

Crucially however, medicine and public health also have anticolonial traditions. Among these traditions are the social medicine movement in Latin America in the 1950s,^32^ as well as various health improvement initiatives in post-independence states that were built on the principles of social justice, equity, community mobilization, culturally appropriate technology and multisectoral action for health.^33^^,^^34^ These initiatives laid the foundation for the World Health Organization’s (WHO) 1978 Alma Ata Declaration,^35^ which arguably remains the exemplary expression of anticolonial global health. Among other things, the Alma Ata Declaration called for a “new international economic order”, “a genuine policy of independence” for developing countries and “peace, détente and disarmament” between nations. Two other anticolonial expressions of global health are: the People’s Charter for Health^36^ developed by the People’s Health Movement, which expresses explicit resistance to neocolonialism within and beyond the health sector; and WHO’s Commission on Social Determinants of Health, which highlighted power asymmetries and unjust and exploitative economic systems as core drivers of health inequalities.^37^

Current discussions on decolonizing global health rarely address the aspirations of the Alma Ata Declaration, the community organizing and empowerment processes exemplified by the People’s Health Movement, or the political economy reforms laid out by the WHO Commission on Social Determinants of Health, all of which are about tackling inequity and injustice in health. To draw from these anticolonial expressions would require an expanded decolonizing global health agenda that would need to address the domination of global health by actors, institutions and knowledge systems in some high-income countries, as well as the undue influence on global health of powerful financial and corporate interests, and their unethical and excessive extraction of wealth through the health sector.^38^^,^^39^ Below we present a three-part agenda for action that can be taken to decolonize global health. 

### Colonialism within global heath

The first part relates to the power asymmetries and unequal relationships that exist between better-resourced and privileged institutions in high-income countries and their counterparts in lower-income countries.^40^ These asymmetries include the structural inequalities in global health education which results in the dominance of universities in high-income countries in global health teaching and research; the financial subsidy of those universities by students from low- and middle-income countries who undertake their education in these colleges; and a contribution to the so-called brain drain from poorer to wealthier countries. Similarly, structural inequalities in global health research produce so-called parachute research (a term used to describe the practice of external researchers dropping into low-income countries and communities for short periods of time to collect research data, and then leaving); and unfair research partnerships, maldistribution of benefits in the form of publications, authorship and citations, kudos or patentable knowledge, and neglect of indigenous knowledge systems and cultures.^41^ Inequitable relationships within global health also manifest in the dependency of poorer countries on external donors and agencies who provide development assistance in ways that fragment and undermine coherent and locally appropriate health systems development,^42^ or that impose the cultural norms of some high-income countries.^43^

An anticolonial agenda within global health must therefore be pursued at two levels. First, actors who are part of relationships and partnerships within global health must be more aware of and sensitive to structural power asymmetries, and must adopt guidelines and codes of conduct aimed at eliminating disparities, preventing unethical practices and instituting mechanisms of mutual accountability.^44^ Second, because aid agencies of high-income countries and private foundations can shape the pattern and nature of relationships within global health, more attention needs to be paid to evaluating the funding and grant-making patterns of these institutions and holding them accountable for perpetuating or worsening the unequal relationships prevalent in the global health system.

### Colonization of global health

The second part relates to the dominance over the structures and systems of global health governance by certain actors and by particular ideas and narratives. This dominance can be traced back to the way powerful actors challenged and undermined the anticolonial vision of the Alma Ata Declaration and then proposed the more conservative vision of selective primary health care.^45^ This dominance can also be seen in the opposition to WHO’s efforts to promote essential medicines lists and the use of generic medicines, and to stop the marketing of breast milk substitutes.^46^

Today, both WHO and the wider global health complex are dominated by wealthy state governments and private foundations largely through their financial power.^47^ In recent decades, public–private partnerships and private financial actors have had increasing influence over global health.^48^ Although multistakeholder models of governance promise greater participation of different stakeholders, these models can also undermine the authority of intergovernmental organizations, while expanding opportunities for powerful private actors to exert influence over governing structures, and concentrating power among parties with less democratic accountability to poorer countries and populations.^49^

Among other things, this situation results in: promotion of selective saleable biotechnological ideas and interventions (often packaged as innovations);^50^ powerful and private actors being shown in a charitable light; global health security discourses that emphasize the protection of wealthy countries and populations from infectious disease threats from poor countries;^51^^,^^52^ and priority-setting exercises that ignore the structural drivers of disease and ill health in poor countries and populations.^53^

An agenda to decolonize global health itself would need to include restoring the authority and capacity to intergovernmental organizations, especially WHO, to coordinate and manage global health as an international concern. At the same time, such an agenda must find ways to make global-level governance more democratic by, for example: enabling the participation of grassroots voices and social movements in global health; improving representation of perspectives from lower-income countries on technical working groups and in global health conferences; and creating new mechanisms to make powerful global health actors more accountable.^54^ Improved diversity and inclusion of different stakeholders in the institutions of global health governance are important. At the same time, however, efforts to overcome underlying power and resource asymmetries will require global health actors to promote fundamental reforms of the political economy aimed at redistributing wealth,^55^ such as those advocated by the WHO Council on the Economics of Health for All,^56^ and the United Nations High-Level Advisory Board on Effective Multilateralism.^57^

### Colonialism through global health

The third part concerns the way exploitation and wealth extraction occurs through the health sector. Although health care is generally benevolent, it is also a trillion-dollar economic sector that creates incentives and opportunities for economic exploitation. The coronavirus disease 2019 (COVID-19) pandemic illustrated this potential for exploitation. The power of pharmaceutical companies and their financial backers, supported by a corporate-friendly system on intellectual property rights, resulted in billions of dollars of profit being generated from a global health emergency that left hundreds of millions of households economically overwhelmed.^38^ The past few decades have seen more parts of the health sector becoming financialized and controlled by a small number of companies, which has created opportunities to extract wealth from public budgets, patients and frontline workers (through downward pressure on wages and increased precariousness in employment conditions).^39^ The control by a few big tech companies of digital health, for example, provides many opportunities to make large profits.^58^ Some of these profits are channelled through tax havens, denying public institutions and services vital revenue.^59^

An agenda to prevent wealth extraction through global health would similarly need to engage with reforms of the political economy aimed at tackling: the under-regulated financialization of the health sector; the abuse of intellectual property rights; the control of key sectors in the health domain by a few oligopolistic corporations; and the high levels of tax avoidance that enable and perpetuate wealth extraction and inequality. Such an agenda may also require global health actors to question their own actions, whether they have tacitly legitimized stakeholders involved in exploitative and extractive practices by including them in health and humanitarian partnerships, or whether they have endorsed charitable projects and philanthro-capitalist models of development that have not been independently and critically evaluated.^60^

## Conclusion

Colonialism manifests in various ways and at different scales. Ultimately, all forms of colonialism are manifestations of power imbalances, and any process of decolonization must therefore challenge how these imbalances are produced and sustained. We have stressed the importance of the financial and corporate forms of contemporary colonialism, while acknowledging the need to address the legacies of the historical territorial and racial forms of colonialism. In doing so, we argue that global health must not only correct historical power imbalances within global health, but also challenge the way global health itself may be colonized, and actively resist unethical and harmful profiteering that can occur through the health economy.

Monopolistic and exploitative markets, harmful marketing and profitmaking, tax abuse, and the use of private wealth to undermine democratic governance and the public interest are the main barriers to freeing global health from historical and contemporary forms of colonialism. Overcoming these challenges calls for a political and economic anticolonialism as well as social decolonization aimed at ensuring greater national, racial, cultural and knowledge diversity within the structures of global health.
